# Effect of Osmotic Pressure on Whey Protein Concentration in Forward Osmosis

**DOI:** 10.3390/membranes11080573

**Published:** 2021-07-29

**Authors:** Pelin Oymaci, Pauline E. Offeringa, Zandrie Borneman, Kitty Nijmeijer

**Affiliations:** Membrane Materials and Processes, Department of Chemical Engineering and Chemistry, Eindhoven University of Technology, P.O. Box 513, 5600 MB Eindhoven, The Netherlands; p.oymaci.akin@tue.nl (P.O.); p.e.offeringa@student.tue.nl (P.E.O.); z.borneman@tue.nl (Z.B.)

**Keywords:** forward osmosis, whey protein, thin-film composite membrane, fouling, osmotic pressure

## Abstract

Forward osmosis (FO) is an emerging process to dewater whey streams energy efficiently. The driving force for the process is the concentration gradient between the feed (FS) and the concentrated draw (DS) solution. Here we investigate not only the effect of the DS concentration on the performance, but also that of the FS is varied to maintain equal driving force at different absolute concentrations. Experiments with clean water as feed reveal a flux increase at higher osmotic pressure. When high product purities and thus low reverse salt fluxes are required, operation at lower DS concentrations is preferred. Whey as FS induces severe initial flux decline due to instantaneous protein fouling of the membrane. This is mostly due to reversible fouling, and to a smaller extent to irreversible fouling. Concentration factors in the range of 1.2–1.3 are obtained. When 0.5 M NaCl is added to whey as FS, clearly lower fluxes are obtained due to more severe concentration polarization. Multiple runs over longer times show though that irreversible fouling is fully suppressed due to salting in/out effects and flux decline is the result of reversible fouling only.

## 1. Introduction

Whey is one of the most abundant dairy by-products obtained from cheese production. For every kg of cheese, on average, 9 L of whey solution is obtained, which results in millions of tons production per year [1]. Formerly treated as waste and used as animal feed, whey has gained high value for human consumption e.g., in baby food and sports, due to its high nutritional value and its high protein content containing all nine essential amino acids, while it is low in carbohydrates and fat. Whey solutions are a by-product of cheese production consisting of water, whey proteins, lactose, minerals and salts. Its composition and pH vary depending on the cheese production source and the whey is classified in four different categories: sweet, native, acidic and salty whey [2,3]. Due to longer shelf life, smaller storage volume and increased transport efficiency, whey proteins are mostly sold as powders rather than as solution. Water removal and drying are thus critical steps in the whey processing process.

Water removal is very energy intensive mostly due to a series of multiple concentrating and drying steps. In conventional processes, water is removed by evaporative concentration steps. Currently the evaporative concentration is often replaced by nanofiltration (NF) and reverse osmosis (RO). Because of the absence of a phase transition, these processes are about 10 times more energy efficient compared to evaporation [4]. However, the increasing osmotic pressure and fouling tendency of the whey solution upon concentrating limit the maximum achievable solid concentration. Compared to NF and RO, forward osmosis (FO) can reach much higher concentration factors because it is less subjective to membrane fouling and less energy demanding [5]. Moreover, since FO is less sensitive to fouling, it requires only little pretreatment. It can also be operated at low temperatures and under low shear conditions making FO very attractive to concentrate high-fouling, heat and shear sensitive streams such as whey protein solutions [4,6].

Aydiner et al. were the first to filtrate whey solutions with an FO integrated membrane system while also taking regeneration of the draw solution (DS) into consideration. Results were compared with a hybrid ultrafiltration (UF)/RO system widely used for whey processing. They showed the technical and economic feasibility of whey production in a sustainable way with FO [7,8,9]. Different types of membrane were investigated including cellulose triacetate (CTA) FO membranes and polyamide (PA) based RO membranes, of which the CTA membranes showed a better performance compared to the RO membranes due to the thinner support structure and lower internal concentration polarization (ICP) [10]. Aydiner et al. investigated the performance of these CTA membranes in more detail considering parameters such as flow rate, pretreatment and DS concentration, emphasizing the adverse impact of the reverse salt (NaCl) flux at DS concentrations above 3 M on the whey solution quality. Seker et al. continued using CTA FO membranes for whey filtration but, as an alternative to NaCl, employed ammonia as draw solution to decrease the reverse salt flux. They suggested further investigation with composite FO membranes to enhance the water flux during whey filtration [11,12]. Recently, Wang et al. employed hollow fiber-based thin-film composite (TFC) FO membranes for whey processing using 0.3, 0.5 and 1 M NaCl DS. The effect of intermediate cleaning with water was investigated using the 0.5 M NaCl DS. It was concluded that a rinsing step with water is sufficient to (partly) recover the flux and to prevent initial fouling and flux decrease [13].

Based on literature, composite FO membranes are preferred giving better performance [5]. Moreover and especially, the reverse salt flux should be as low as possible, as high reverse salt fluxes operate at the expense of product quality and deterioration of the whey [14]. Simultaneously, water fluxes and associated concentration factors should be increased for economic viability [6].

The concentration gradient between the feed and the draw solution (i.e., over the membrane) is the main parameter determining the water flux and thus the concentration factor, but simultaneously also influences the reverse salt flux: a high concentration gradient (high driving force) results in high water fluxes but also adversely impacts the reverse salt flux.

With this in mind, we systematically investigate the effect of driving force on the water flux and reverse salt flux and the specific reverse salt flux (ratio of both fluxes) using a TFC FO membrane. NaCl is used in the draw solution to concentrate a whey solution. We not only vary the concentration gradient over the membrane by varying the concentration of the DS, but also by changing both the concentration of the feed solution (FS) and the DS in order to have a similar concentration gradient at different absolute concentrations of the two solutions. Not only the process performance in terms of flux and concentration factor is investigated, but also the effect of membrane fouling.

## 2. Materials and Methods

### 2.1. Materials

Commercial thin-film composite membranes were obtained from Bluetec (Renkum, The Netherlands). Sodium chloride (Sanal P^®^) was kindly supplied from Nouryon (Deventer, The Netherlands). Ultrapure water (UPW) was produced using an ELGA Purelab (VWS, High Wycombe, UK) water purification system (18.2 MΩ·cm) and used to prepare all solutions. Organic whey protein powder from Purasana (Ypres, Belgium) (natural, unflavored, without additives, composition given in Table 1) was used to prepare the whey solutions. Isopropanol was supplied by VWR Chemicals (Radnor, PA, USA). All chemicals were used as received. All solutions were used at ambient pH without any adjustment.

### 2.2. Whey Protein Solution Preparation

Whey protein solutions with a composition as shown in Table 2 were prepared by stirring whey protein powder in UPW (8.75 g whey powder for 1 L solution). The composition given in Table 2 resembles sweet whey except for the lower lactose and salt concentrations in order to focus on the effect of whey proteins rather than on other components during concentration [2,3]. The osmotic pressure of the whey protein solution is estimated to be 0.1 bar, using the Van’t Hoff equation.

Salty whey protein solutions were prepared by dissolving, under stirring, whey protein powder (8.75 g/L) and 0.5 M NaCl in UPW. With this salt content, the composition resembles salty whey solutions that are obtained after salty cheese production [3]. The osmotic pressure of the salty whey protein solution is around 23 bar (osmotic pressure of 0.5 M NaCl solution).

Draw solutions were prepared by dissolving NaCl in UPW according to the desired concentrations. The osmotic pressure of the NaCl solutions was calculated with Aspen Plus^®^ software (Bedford, MA, USA) to account for the high NaCl concentrations of the DS deviating from ideality. The software employs the Pitzer model involving the water activity to calculate the osmotic pressure of highly concentrated solutions. 

### 2.3. Membrane Pretreatment

TFC membranes were pretreated before use by immersing the membranes in isopropanol for half an hour, followed by a rinsing step with UPW.

### 2.4. Membrane Characterization

#### Forward Osmosis Performance

FO filtration measurements were conducted on a crossflow FO filtration system. A membrane cell (Convergence Industry B.V., Enschede, The Netherlands) with two slits located on both ends of the membrane was used to hold the membrane with an effective membrane area of 0.006 m^2^ (40 mm width, 150 mm length) and 5 mm slit height. The membrane was mounted with the active layer facing the feed solution (FO mode). Diamond shaped spacers with a thickness of 2 mm were used, two on each side of the membrane. UPW, 0.5 M NaCl or the whey protein solution with or without 0.5 M NaCl was used as feed solution (FS) (1.8 L) and 1 L NaCl solution was used as draw solution with varying initial concentrations of NaCl as shown in Table 3.

The feed and draw solutions were circulated in the system by two diaphragm pumps (Liquiport, KNF, Freiburg im Breisgau, Germany). The co-current flow rates were set to 36 ± 2 L/h giving a crossflow velocity of 5.7 cm/s (assuming 85% void space). Measurements were performed in batch mode at ambient temperature and continued for around 6 h for whey or salty whey protein concentration. Prior to concentration of the whey protein solutions without NaCl, first, the UPW flux of a membrane was measured. After that, the whey feed solution was introduced. After each whey run, membranes were rinsed for half an hour with demineralized water for cleaning to remove possible reversible fouling. After cleaning, an additional short run with UPW was done to measure the UPW flux of the membrane after use to compare it to the UPW flux of the unused membrane. In case of concentration of the salty whey protein solution, first 0.5 M NaCl FS without whey was used to measure the flux in the presence of salt in the FS. Next, 0.5 M NaCl with whey as FS was introduced. After each salty whey run, membranes were again rinsed for half an hour with demineralized water to remove reversible fouling. After cleaning, a short run with 0.5 M NaCl solution without whey was done to measure the water flux after use of the membrane and compare these flux values to those of the unused membrane. For all the experiments, at each run fresh solutions were used for both feed and draw solutions.

The water flux J_w_ (L/m^2^ h) was calculated from the collected mass of permeate volume in the draw solution V_d_ (L) in a certain time t (h) per membrane area A (0.006 m^2^) (Equation (1)). The change in conductivity in time was measured at the feed side and used to calculate the reverse salt flux J_s_ (g/m^2^ h) from the change in salt concentration c (g/L), the feed solution volume V_f_, the time elapsed and the membrane area (Equation (2)).

(1) $$J_{w}=\;\frac{V_{d}}{A\cdot t}\;,$$

(2) $$J_{s}=\frac{c\cdot V_{f}}{A\cdot t},$$

The specific reverse salt flux was calculated as the ratio of the reverse salt to water flux J_s_/J_w_ (g/L). The concentration factor was calculated by dividing the initial volume of the FS to the final volume after concentration.

## 3. Results and Discussion

### 3.1. Membrane Performance

#### 3.1.1. Ultrapure Water Feed Solution

**Figure 1:**
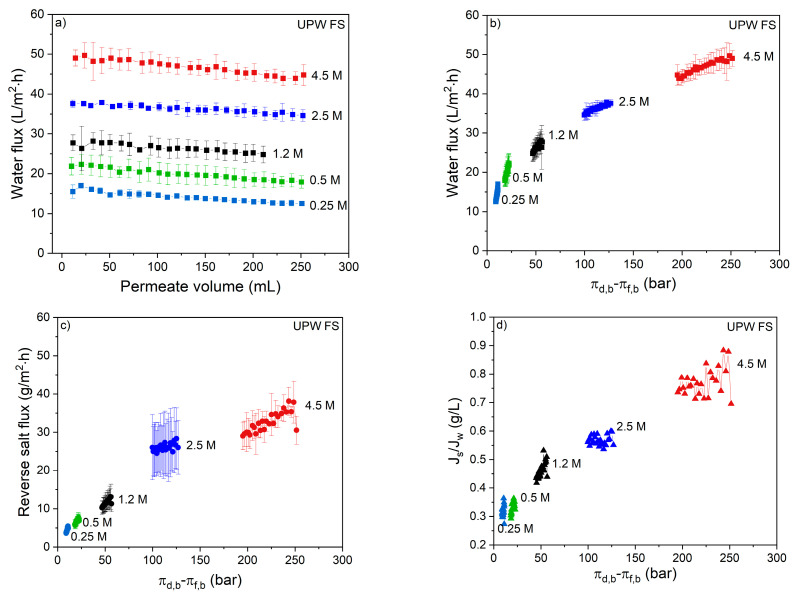
Membrane performance with various DS concentrations and UPW as FS in terms of (**a**) water flux as a function of permeate volume and (**b**) osmotic pressure difference; (**c**) reverse salt flux as a function of osmotic pressure difference and (**d**) specific reverse salt flux as a function of osmotic pressure difference between bulk DS and FS. Average values represent two runs.

The membrane performance was first investigated using UPW as FS and different NaCl concentrations as DS. The effect of DS concentration on the water flux can be seen in Figure 1a.

**Figure 2:**
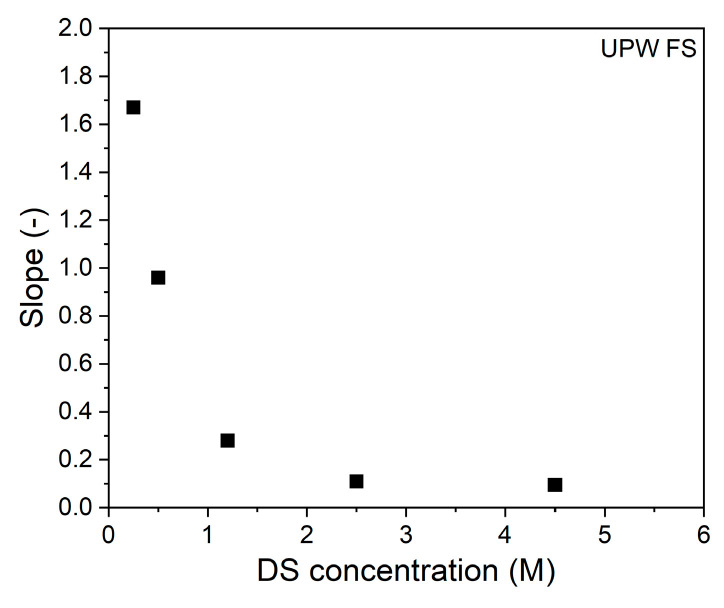
Slopes of the water flux vs. osmotic pressure difference between bulk DS and FS at each DS concentrations. FS: UPW.

During the FO process, water permeation dilutes the draw solution thereby reducing the osmotic gradient, resulting in a reduced water flux with permeated volume (Figure 1a). For the used volume of 1 L DS, this means that 250 mL of water permeation dilutes the DS with 20%. In the same time interval, the reversed salt flux increases the osmotic pressure of the feed solution by 0.04 till 0.12 bar for the 0.25 and 4.5 M DS, respectively (Figure 1b,c). This implies that for all DS solutions the osmotic pressure is 2–3 orders of magnitude higher than that of the FS. This proves that the effective osmotic pressure and its decrease during the progression of the process is fully dictated by the DS concentration and its dilution. The water flux is not proportional to the osmotic pressure of the DS due to internal concentration polarization (ICP; Figure 1b). The same behavior was also observed by Tang et al. [15], showing that severe ICP occurs at high DS concentrations because of the relatively high reverse salt flux in relation to the water flux. It is important to realize that, relatively, the reverse salt flux increases more with the DS osmotic pressure than the water flux. The consequence of this is that, when the reversed salt flux is detrimental for the quality of the concentrated feed product, lower salt concentrations in the DS are preferred above higher salt concentrations in the DS (Figure 1d). From Figure 1b, the slope of the data at each specific DS can be calculated. These values are reported in Figure 2.

Clearly, the slope of the curve decreases with increasing DS concentration. In other words, the available osmotic pressure difference is less effectively used at higher DS concentrations. Of course, as the absolute concentration gradient over the membrane is higher at higher DS concentrations, the process can operate longer and, in time, more water can be removed when a higher DS is applied.

#### 3.1.2. Whey Protein Feed Solution

**Figure 3:**
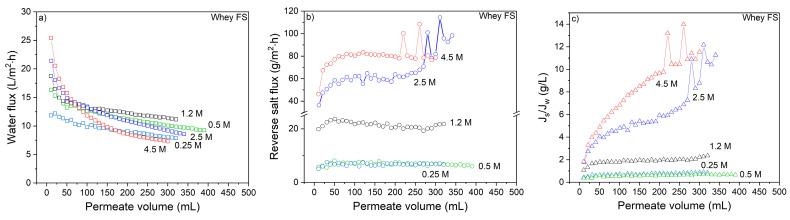
The effect of draw solution concentration on the (**a**) water flux; (**b**) reverse salt flux and (**c**) specific reverse salt flux during whey filtration as a function of permeate volume. FS: 8.75 g/L whey; Osmotic pressure: 0.1 bar. Average values represent two runs.

Next, experiments with whey solutions as feed were performed. Figure 3a shows the change in water flux using different DS concentrations to concentrate the whey solution.

Compared to the experiments with UPW as feed, whey proteins in the FS cause a sharp initial decline in the water flux (compare Figure 1a with Figure 3). This sharp initial decline is followed by a gradual decline in water flux that can be attributed to the dilution of the DS. The initial decline points out that instantaneous protein deposition occurs on the surface of the membrane that decreases the water flux. This initial flux decline is considered to be only due to the membrane fouling, considering the negligible change in osmotic pressure of both DS and FS solutions at the initial stages. During the first 100 mL of permeation the flux decline compared to the initial flux is 33%, 43%, 45%, 63% and 72% for the 0.25, 0.5, 1.2, 2.5 and 4.5 M NaCl DS, respectively, showing that more severe flux decline occurs at higher DS concentrations.

Remarkably, contrary to FO with UPW as FS, the order of flux values does not align with the draw solution concentration. The water flux increases with the DS concentration up to 1.2 M, followed by a decrease in water flux using higher DS concentrations. Moreover, the reverse salt flux also increases as the DS concentration increases above 0.5 M (Figure 3b). At lower DS concentrations, the reverse salt flux values are similar to that of UPW as FS. However, at higher DS concentrations, reverse salt fluxes double when UPW was used as FS. The calculation of the reverse salt flux during whey filtration neglects the initial conductivity value of the whey protein solution itself. When whey is present in the FS, the feed conductivity increase is higher compared to when UPW is used as FS (Table S1). Therefore, conductivity values of the whey protein FS at the initial and final stages are also given in the Table S2. Counterintuitively, this is due to the dramatic decrease in water flux during whey concentration. Due to the lower water flux, also the drag forces associated with this water flux decrease. High drag forces prevent excessive salt transport from DS to FS. Lower drag forces thus give rise to higher reverse salt fluxes. This not only obvious when the conductivity changes of UPW and whey as FS are compared, but also when the low (0.25–1.2) and high (2.5 and 4.5) concentration DS solutions are compared. The initial increase of reverse salt flux is also aligned with the initial water flux decline observed in Figure 3a. The initial incline in reverse salt flux also increases further as DS concentrations increase from 1.2 to 2.5 and 4.5 M. This strengthens the explanation above, showing that, as soon as the water flux starts decreasing, the reverse salt flux increases as the drag force diminishes. As a result of this, the specific reverse salt flux increases at the same time (Figure 3c), emphasizing the adverse interaction between water and reverse salt flux with values increasing above 1 g/L at DS concentrations higher than 1.2 M NaCl.

A possible reason for the decrease in water flux using high DS concentrations is that more whey protein accumulation on the membrane surface takes place at higher DS concentrations, also due to the increased dragging force as a result of higher water fluxes, but also due to the higher reverse salt flux at high DS concentrations that results in protein salting out. Salting out occurs when the water molecules in the feed solution are no longer able to surround the charges of the ions and proteins [16,17]. This enhances the hydrophobic interactions between protein molecules, ultimately leading to aggregation and subsequently to denaturation and precipitation of proteins. Protein precipitation follows the Hoffmeister series where the anion concentration has a stronger effect on the precipitation [17]. This process starts close to the membrane surface since the protein concentration is the highest there due to water transport and external concentration polarization (ECP), and at the same time the boundary layer is also the location where the salt concentration is the highest because of the reversed salt flux that permeates through the membrane. This effect is most pronounced at high DS concentrations, since then the initial water flux (associated with ECP) and the reverse salt flux are the highest. This increase causes a sharp loss in water flux almost immediately after the start of the experiment. However, this loss can then partly be mitigated by lower ICP as a result of low water flux and the decline becomes more gradual [18]. The NaCl concentration in the FS depends on the reverse salt flux which then can change the protein solubility due to the salting in/out effects. It is reported that Na ions minimize the intermolecular repulsion of unfolded proteins that results in agglomeration due to the attraction and creation of a protein network [19]. On the other hand, Cl ions are found to bind to the proteins due to their weaker hydration affinity. That is also the reason why low NaCl concentrations can be used as a protein cleaning agent to remove bound whey proteins from a membrane surface [20]. However, the salt concentration at the membrane surface is expected to be higher compared to the concentration in the bulk of the solution which can thus induce protein precipitation at the membrane surface. Section 3.1.3 investigates this effect of salting out in relation to the reverse salt flux in more detail.

Next, Table 4 reports the obtained whey solution concentration factors at different DS concentrations.

The highest concentration factor of the FS (1.3) is obtained with the 1.2 M NaCl DS after 5.5 h of filtration time. The flux values are higher during a longer period of time with a slower decrease compared to the other DS concentrations. Aydiner et al. [9] showed a concentration factor of ~2.14 in 6 h for a CTA FO membrane (140 cm^2^), a 3 M NaCl DS and whey as FS (both 3 L initial volume) with a water flux decrease from 25 to 14 L/m^2^ h. Even though the DS concentration was as high as 3 M NaCl, the reverse salt flux value reported was 4.8 g/m^2^ h, which is lower than the values reported here. This lower reverse salt flux results in slower decline in osmotic pressure and water flux and thus lower salt concentrations in the FS, less deterioration the properties of the whey after concentration and less salting out of proteins. Wang et al. [13] employed a TFC hollow fiber-based FO membrane (106 cm^2^) to concentrate a whey solution (3 L) with a 0.5 M NaCl DS (8 L). At the end of an 8 h cycle, a concentration factor of 1.5 was reached with the flux dropping by 10% relative to the initial flux of 11.7 L/m^2^ h. In addition, this membrane had a reverse salt flux of ~3 g/m^2^ h using a 10 mM NaCl FS and a 0.5 M NaCl DS. Comparing these results, the flat-sheet TFC membrane has a higher reverse salt flux and strengthens our further investigation on the effect of reverse salt flux on the decrease in water flux due to the change in protein solubility (salting out).

**Figure 4:**
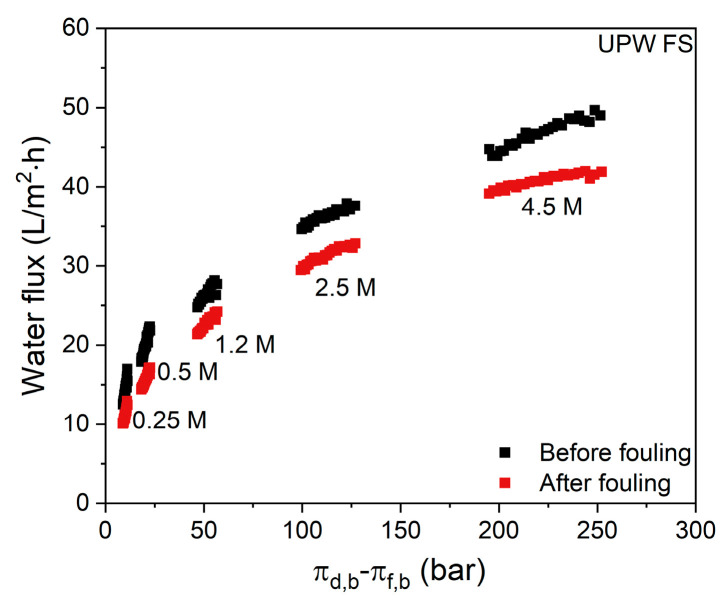
Water flux values before and after the first cycle with whey proteins with various NaCl concentration. FS: UPW. Average values represent two runs.

To visualize the effect of protein fouling on the membrane, the water flux with UPW as FS of a native membrane and that of the same membrane after whey concentration is compared in Figure 4. Before the flux of the fouled membrane is measured, it is gently flushed with clean water to remove loose and residual proteins.

For all used DS (NaCl) concentrations the water flux value was restored to about 85% of the initial water flux of the native membrane after the membrane was deployed for 6 h to concentrate whey proteins. This shows that the fouling layer that is formed on the membrane surface can to a large extent be removed by water cleaning only and that the severe initial flux decline during whey concentration is, thus, to a large extent the result of reversible fouling, concentration polarization and salting out effects, as will be discussed in more detail.

#### 3.1.3. Salty Whey Protein Feed Solution

**Figure 5:**
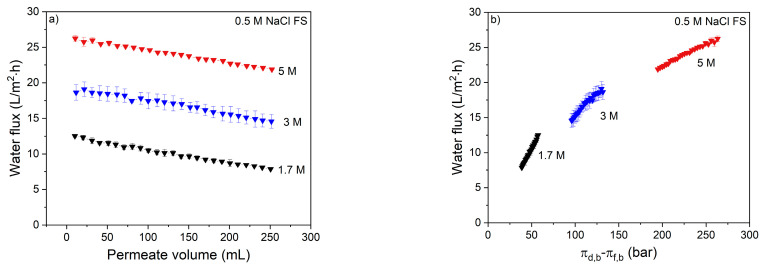
The effect of osmotic pressure difference with 0.5 M NaCl in the FS on (**a**) the water flux as a function of permeate volume and (**b**) the water flux as a function of effective osmotic pressure (FS: 0.5 M NaCl). Average values represent two runs.

Next, experiments with additional salt (0.5 M) added to the FS are performed. In this way the combined effect of concentration gradient (i.e., driving force) over the membrane and the absolute salt concentrations of both FS and DS can be decoupled and both can be investigated separately. The concentration of the DS is set at 1.7, 3 or 5 M to maintain a similar concentration difference over the membrane as in the previous experiments (1.2, 2.5 or 4.5). The corresponding water fluxes are reported in Figure 5. Due to the higher conductivity of the 0.5 M NaCl in the FS, the change in conductivity of the feed solution upon concentration could not be measured accurately. Therefore, reverse salt flux and specific reverse salt flux of the 0.5 M NaCl FS and 0.5 M NaCl with whey FS could not be calculated and are thus not reported.

**Figure 6:**
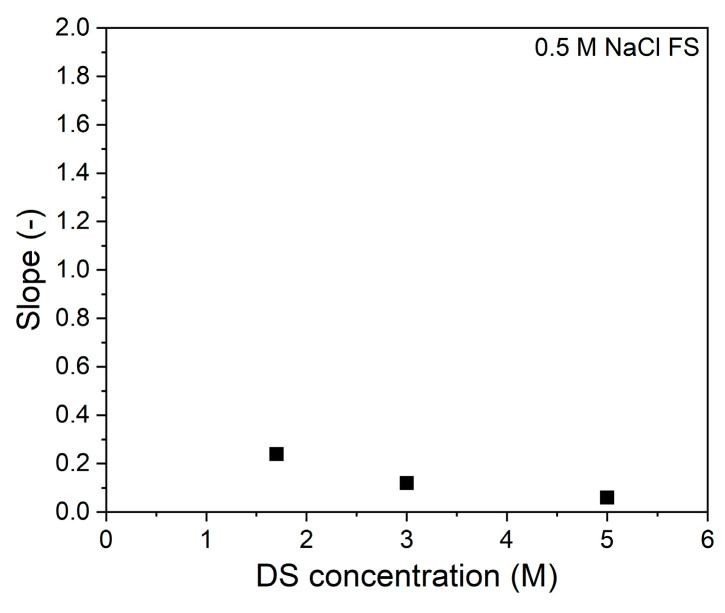
Slopes of the water flux vs. osmotic pressure difference between bulk DS and FS at each DS concentrations. FS: 0.5 M NaCl FS.

Clearly, when 0.5 M NaCl is added to the FS, the initial water flux decreases compared to the water fluxes with 100% UPW FS, even though the concentration differences are the same. This is due to ECP that occurs at the active layer side of the membrane [21]. ECP decreases the effective osmotic pressure difference over the membrane, in addition to ICP, and further decreases the flux values. This cannot be compensated for by the increased DS concentration, as that induces stronger ICP [21], also decreasing the effective concentration gradient (the DS faces the porous support side). From Figure 5b, the slope of the data at each specific DS is also calculated and shown in Figure 6.

Similar to Figure 2, here the slope of the curves decreases as the DS concentration increases. This shows that increasing the osmotic pressure becomes less beneficial for gaining water flux, due to increased ICP and ECP.

**Figure 7:**
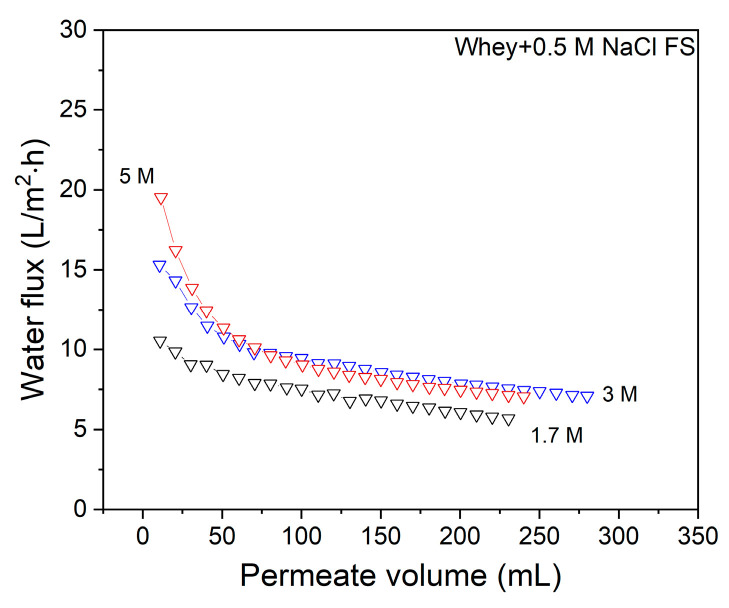
The effect of draw solution concentration on the water flux during the FO filtration of whey solution with 0.5 M NaCl. FS: 8.75 g/L whey + 0.5 M NaCl solution. Average values represent two runs.

Next the effect of the presence of whey in the FS with an additional 0.5 M NaCl added is investigated for different DS concentrations (Figure 7).

**Figure 8:**
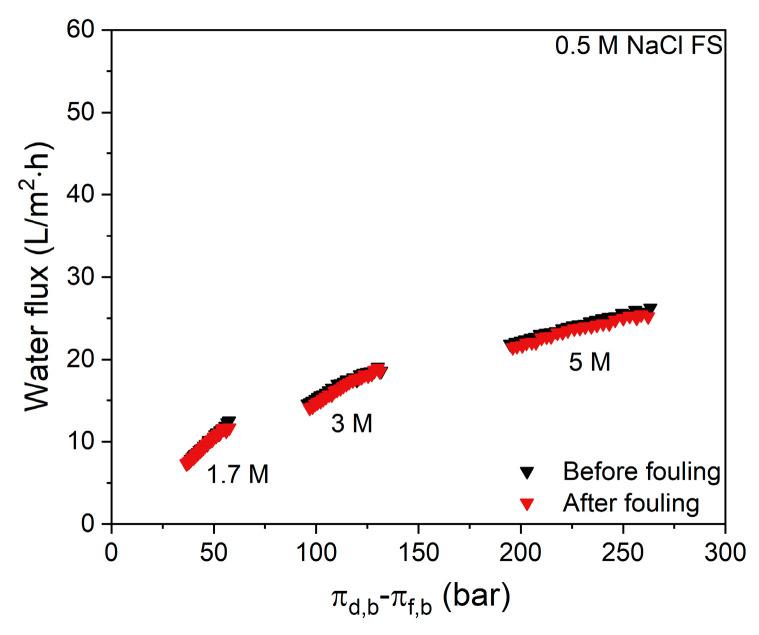
Water flux values before and after the first cycle of salty whey filtration with various NaCl concentrations in the DS. FS: 0.5 M NaCl. Average values represent two runs.

When additional salt is added to the whey FS, the initial flux values for all DS concentrations (Figure 7) are further decreased when compared to the flux values in Figure 3 where no NaCl is added to the whey protein solution. Although the flux decline in time seems to be somewhat slower for all DS concentrations when 0.5 M NaCl is added to the FS, the overall effect is that salt addition to the FS worsens the water flux performance. This results in low concentration factors of 1.15, 1.20 and 1.20 for 1.7 M, 3 M and 5 M NaCl DS, respectively. This observation is especially important for whey solutions that are obtained after productions of salty cheese types, such as Cheddar, which contains up to 1.7 M NaCl in the solution [3]. Similar to Figure 4, now water flux values of the native membrane and that of a membrane gently cleaned with water after whey filtration are also reported (Figure 8).

For all DS concentrations, there is no significant difference in flux value before and after whey filtration. Osmotic pressures of some concentrations of NaCl solutions are given in Table S3. Comparing these results with the data in Figure 4 shows that, even though the water flux values are slightly lower, a persistent fouling layer is not observed when NaCl is added to the FS, which will be discussed in more detail in the next section.

#### 3.1.4. Time-Dependent Performances

**Figure 9:**
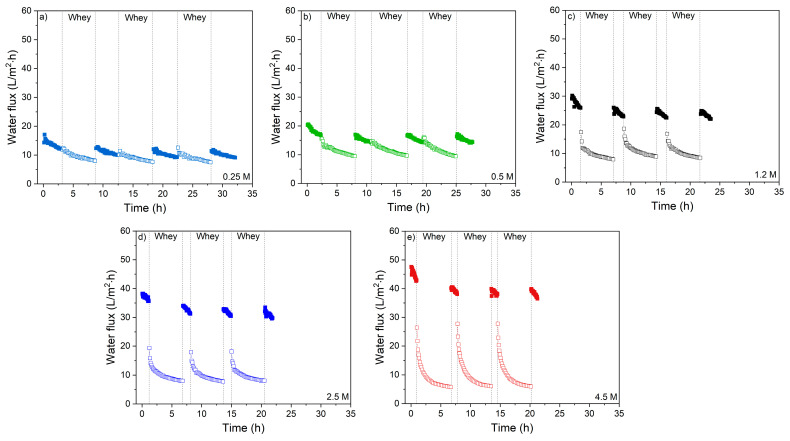
Water flux of the membranes in longer periods of whey filtration (three times, in total ~17 h) with intermediate rinsing and measuring with UPW FS with (**a**) 0.25 M; (**b**) 0.5 M; (**c**) 1.2 M; (**d**) 2.5 M and (**e**) 4.5 M NaCl DS concentrations.

**Figure 10:**
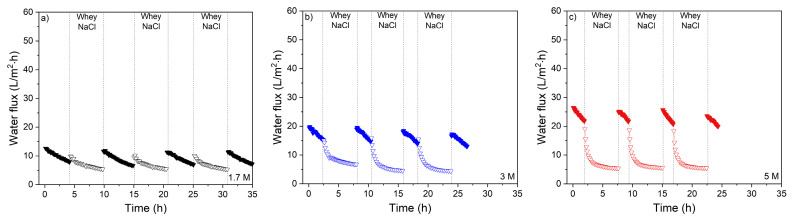
Water flux of the membranes in longer periods of salty whey filtration (three times, in total ~17 h) with intermediate rinsing with UPW and measuring with 0.5 M NaCl FS with (**a**) 1.7 M; (**b**) 3 M and (**c**) 5 M NaCl DS concentrations.

To evaluate the membrane performance in sequential runs, multiple whey concentration cycles were performed, each cycle intermitted by an UPW cleaning step. First data for whey without salt addition are reported (Figure 9), followed by the data obtained for salty whey (Figure 10).

In all cases, obviously a clean membrane with UPW as feed outperforms the used membranes for all DS concentrations. The flux decreases dramatically during the first whey protein filtration. After the first rinsing cycle, the water flux restores to about 85% for all DS concentrations and stays constant in the sequential cycles, indicating that only a small amount of the fouling is caused by irreversible fouling that cannot be removed with UPW only. This suggests that only a thin and relatively open fouling layer remains at the membrane surface after gentle UPW cleaning. During the sequential whey protein filtration cycles, usually a reversible fouling layer grows causing flux decline, while the contribution of irreversible fouling remains reasonably constant.

Next, Figure 10 shows the cyclic water flux in time of three salty whey protein solutions cycles (with 0.5 M NaCl in the FS) using 1.7, 3 and 5 M NaCl as DS intermitted by UPW cleaning.

Multiple runs with salty whey solutions show similar behavior. In agreement with Figure 8, the water flux is restored after UPW cleaning for almost 100%. A small decrease in water flux only occurs after multiple successive cycles, showing minor irreversible fouling. This is in contrast to Figure 9, where an initial drop in water flux is observed showing irreversible fouling. The presence of salt in the salty whey FS results in salting out of proteins. Moreover, the lower drag force due to the lower water fluxes cause ECP. Due to the increased salt concentrations, proteins cluster, forming larger foulants [17] and, due to salting out effects, proteins precipitate and adsorb on the membrane surface. Lower flux values and therefore lower drag force on foulants might cause less adsorption at the same time, which is irreversible. Moreover, this effect can be strengthened by the presence of larger foulants due to salting out of proteins that alters the adsorption on the surface. This suggests that NaCl reduces irreversible fouling whereas, in the absence of NaCl, irreversible fouling occurs instantaneously decreasing the flux in the subsequent runs. During salty whey concentration, especially reversible fouling causes a sharp decline in flux. Comparing similar flux values of whey FS without or with NaCl (e.g., 0.5 M DS-whey FS and 3 M DS-salty whey), it is clear that drag forces can be neglected and that the salting out effect is responsible for most of this behavior.

## 4. Conclusions

The effect of the concentration gradient over the membrane on the process performance of a thin film composite (TFC) membrane for the concentration of whey was investigated. Experiments with UPW as feed and various draw solution concentrations show a higher flux with increasing total amount of water that permeated the membrane. A higher osmotic pressure gradient results in a higher water flux. When whey instead of UPW is used as feed solution, the flux initially decreases very quickly due to instantaneous protein fouling of the membrane. At higher concentrations of the draw solution, this flux decline is more severe. The reverse salt flux also increases at higher draw solution concentrations. This is due to the lower water fluxes and, along with this, lower drag forces of the water on the salt permeating in opposite directions. The decrease in flux is mostly due to reversible fouling, and to a smaller extent to irreversible fouling. Concentration factors in the range of 1.2–1.3 are obtained. In all cases, gentle cleaning with ultrapure water recovers the UPW flux to at least 85% of the initial value. When high product purities are required, operation at lower draw solution concentrations and low reverse salt fluxes are required.

Experiments with an additional amount of 0.5 M NaCl added to the whey feed solution show that at similar concentration gradients over the membrane, water fluxes are lower due to external and internal concentration polarization. Multiple runs over longer times show that, in that case, irreversible fouling is fully suppressed due to salting in/out effects and flux decline is the result of reversible fouling.

## Figures and Tables

**Table 1:** Composition of whey protein powder provided by the manufacturer.

Component	g Per 100 g
Protein	80
Sugar	3.5
Fat	5
Salt	0.5
Moisture	11

**Table 2:** Whey protein solution composition used as feed solution.

Component	g Per 100 mL
Protein	0.7
Sugar	0.03
Fat	0.04
Salt	0.004

**Table 3:** FO test conditions.

Feed Solution	Draw (NaCl) Solution Concentration (M)				
UPW	0.25	0.5	1.2	2.5	4.5
Whey	0.25	0.5	1.2	2.5	4.5
0.5 M NaCl			1.7	3.0	5.0
Whey + 0.5 M NaCl			1.7	3.0	5.0

**Table 4:** Concentration factors at different DS concentrations after 5.5 h of FO operation.

DS Concentration (M)	Concentration Factor (-)
0.25	1.21 ± 0.01
0.5	1.26 ± 0.00
1.2	1.30 ± 0.11
2.5	1.25 ± 0.04
4.5	1.22 ± 0.04

## Data Availability

The data presented in this study are available on request from the corresponding author.

## Supplementary Materials

The following are available online at https://www.mdpi.com/article/10.3390/membranes11080573/s1, Table S1: Feed UPW solution conductivities of initial and final stages after 1 h of operation at different DS concentrations, Table S2: Feed whey solution conductivities of initial and final stages after 5.5 h of operation at different DS concentrations, Table S3: Osmotic pressure values of various concentrations of NaCl solution.
