# Supplementary material for: Effect of Osmotic Pressure on Whey Protein Concentration in Forward Osmosis
Source: Membranes (Basel). 2021 Jul 29;11(8):573. doi: 10.3390/membranes11080573 (PMC8401517; doi:10.3390/membranes11080573)
Supplement: Supplementary file 1 [file membranes-11-00573-s001.zip › membranes-1323338-supplementary.pdf]

membranes

MDPI

Supporting Information

Effect of Osmotic Pressure on Whey Protein Concentration in Forward Osmosis

Pelin Oymaci, Pauline E. Offeringa, Zandrie Borneman and Kitty Nijmeijer *

Membrane Materials and Processes, Department of Chemical Engineering and Chemistry, Eindhoven University of Technology, P.O. Box 513, 5600 MB Eindhoven, The Netherlands; p.oymaci.akin@tue.nl (P.O.); p.e.offeringa@student.tue.nl (P.E.O.); z.borneman@tue.nl (Z.B.)

* Correspondence: d.c.nijmeijer@tue.nl; Tel.: +31-40-247-2871

Table S1. Feed UPW solution conductivities of initial and final stages after 1 hours of operation at different DS concentrations.

Initial DS Concentration (M)	FS conductivity (µS/cm)	
	Initial	Final
0.25	3.7 ± 0.0	33.6 ± 1.3
0.5	4.5 ± 0.3	48.7 ± 5.9
1.2	7.1 ± 1.8	80.5 ± 15.7
2.5	12.4 ± 2.5	179.5 ± 47.4
4.5	11.1 ± 0.8	224.5 ± 21.9

Table S2. Feed whey solution conductivities of initial and final stages after 5.5 hours of operation at different DS concentrations.

Initial DS Concentration (M)	FS Conductivity (µS/cm)	
	Initial	Final
0.25	192 ± 10	481 ± 13
0.5	187 ± 6	503 ± 74
1.2	243 ± 13	1155 ± 191
2.5	257 ± 18	3265 ± 926
4.5	217 ± 33	3180 ± 127

Table S3. Osmotic pressure values of various concentrations of NaCl solution.

NaCl Concentration (M)	Osmotic Pressure (bar)
0.11	5.1
0.50	22.5
1.00	46.3
1.49	70.8
2.04	100.5
2.53	128.9
3.03	158.8
3.52	189.9
4.02	222.1
4.51	255.1
5.01	288.9
